# Coating Process of Oil and Gas Well Pipeline Preventive Repair Materials Inspired by Remora Suckerfish Structure

**DOI:** 10.3390/biomimetics10070436

**Published:** 2025-07-02

**Authors:** Yuliang Lu, Dongtao Liu, Jiming Song, Qiaogang Xiao, Kezheng Du, Xinjie Wei, Lifeng Dang, Yajun Yu, Huiyan Zhao

**Affiliations:** 1 CNOOC Ener Tech-Drilling & Production Co., Shenzhen 518067, China; luyl@cnooc.com.cn (Y.L.); songjm2@cnooc.com.cn (J.S.); xiaoqg@cnooc.com.cn (Q.X.); dukzh@cnooc.com.cn (K.D.); weixj6@cnooc.com.cn (X.W.); danglf2@cnooc.com.cn (L.D.); 2 School of Biological and Agricultural Engineering, Jilin University, Changchun 130022, China; huiyan24@mails.jlu.edu.cn

**Keywords:** oil and gas well pipelines, preventive rehabilitation, bionics, remora suckerfish, discrete element method, interfacial properties

## Abstract

To meet the special needs of preventive maintenance for oil and gas well pipelines, this study conducts a geometric dissection of remora suckerfish based on bionics. It combines the biological features with fiberboard tape and uses the discrete element method to construct a particle model of solvent-free, epoxy-reinforced polymer materials, determining relevant parameters. The model accuracy is verified through volumetric density and drop tests, and the optimal parameter combination of the remora-inspired structure is obtained via multi-factor simulation analysis. Comparative tests confirm that the bionic structure enhances stability by approximately 43.29% compared to the original structure, effectively avoiding insufficient strength. It successfully addresses the gravitational segregation and fluid shear caused by uneven coating thickness, ensures stable and reliable interfacial properties of the composite structure during service, and provides strong support for the practical application of related materials in the preventive repair of oil and gas well pipelines. The findings promote the upgrade of oil and gas pipeline maintenance strategies from “passive response” to “active prevention”, laying the core technical foundation for the resilience of energy infrastructure.

## 1. Introduction

As an important infrastructure of the energy industry, oil and gas well pipelines have been facing complex service environments such as high temperatures, high pressure, corrosive media erosion, and mechanical wear for a long time, and their failure is mainly manifested as corrosion perforation, crack extension, and structural deformation [1,2,3,4,5]. The traditional repair process relies on spraying, roller coating, and other coating technologies, but there are defects such as insufficient coating bonding strength, poor uniformity, and short service life, especially in the casing joints, watertight casing splash zone, and other critical parts, meaning that long-lasting protection is difficult to realize [6,7,8]. To address this technological bottleneck, this study explores the biomimetic application of remora suckerfish structure in the design of pipe repair materials from the perspective of biological evolution.

Remora suckerfish achieve high adsorption efficiency through their specialized sucker structure, which consists of a synergistic combination of fins, bony spicules, and labial ring tissues, and produces strong interfacial bonding through negative pressure adsorption and mechanical interlocking mechanisms [9]. Studies have shown that this biostructure can increase the interfacial shear strength by more than 30%, which is significantly better than the bonding effect of traditional adhesives [10,11]. In engineering, biomimetic structures are more widely used. Wang et al. [12] used a biomimetic remora suckerfish underwater robot to achieve strong adhesion and hitchhiking on various surfaces including smooth, rough, and submissive surfaces, as well as sharks’ skin. Wang et al. [13,14] investigated the effects of remora suckerfish suction cups’ soft-lip material, pretension, and lamellar motion on tangential friction under different adhesion states (forward and backward) effects. However, the bonding behavior of other materials remains underexplored, and optimizing the bionic structure design is essential to assess its application prospects for different research targets.

It is worth noting that the traditional experimental method requires a large number of trial-and-error experiments when optimizing the bionic structure by changing its geometrical parameters, which leads to a long research and development cycle and high costs. The discrete element method is a numerical computation method used to simulate the behavior of discrete particle systems, which can intuitively simulate the microscopic behaviors and macroscopic phenomena of particle systems and reveal the interaction mechanisms between particles [15,16,17,18]. For example, Jin et al. [19] modeled the dynamic loading problem of granite residual soil when it is used as a subgrade fill for pavement engineering by means of the discrete element method. Hao et al. [20] numerically investigated a three-dimensional conical nozzle bed by using CFD (Computational Fluid Dynamics) and the discrete element method and systematically analyzed the particle–gas-flow pattern, bubble volume fluctuation, and fountaining characteristics. Sun et al. [21] took into account the formation’s particle size distribution (PSD) characteristics, and a complex particle-size particle injection model was established by a coupled CFD-DEM (Discrete Element Method) method. In the field of preventive rehabilitation of oil and gas well pipelines, the homogeneity and bond strength of coating materials directly determine the service life and safety performance of pipelines [22,23]. Currently, researchers are working to improve these two key properties through various ways. In experimental studies, it is common to adjust the material formulation, for example, by adding nanoparticles [24] or coupling agents [25] to improve the internal compatibility of the coating and enhance the bonding effect; in theoretical studies, finite element simulation is widely used to analyze the interfacial stress distribution and guide the design of the coating structure [26]. However, there are still limitations in the existing methods, for instance, nano-additives may increase the material cost and preparation complexity and simulation studies are mostly based on ideal conditions, which deviate from the actual working conditions. Therefore, it is urgent to develop a low-cost, easy-to-use method that can solve the homogeneity and bonding problems at the structural root. In this study, we innovatively introduced discrete element numerical simulation technology to systematically analyze the influence law of bionic structure parameters on interfacial adhesion performance by establishing a multi-scale contact mechanics model, breaking through the limitations of traditional experimental methods.

Based on the principle of bionics, this paper analyzes the geometric structure of remora suckerfish and integrates its unique configuration with fiberboard tape for the preventive repair of oil and gas well pipelines. Then, the particle model of the polymer material is constructed by discrete element method, and the related parameters are precisely determined. The accuracy of the model was verified by a solvent density test and a drop test. With the help of multi-factor test simulation analysis, the optimal combination parameters of the bionic structure were determined. On this basis, the comparison test between the bionic structure and the original structure is carried out, which clarifies the stability of the bionic structure and effectively avoids the problems of insufficient strength that may exist in the original structure. This method avoids the uneven coating thickness caused by the gravitational segregation of polymer materials and fluid shear. It ensures the composite structure maintains stable and reliable interfacial performance during service. This lays the foundation for the practical application of related materials in the preventive repair of oil and gas well pipelines.

## 2. Materials and Methods

### 2.1. Structural Characteristics and Adsorption Mechanisms of Remora Suckerfish Remora Suckers

**Figure 1:**
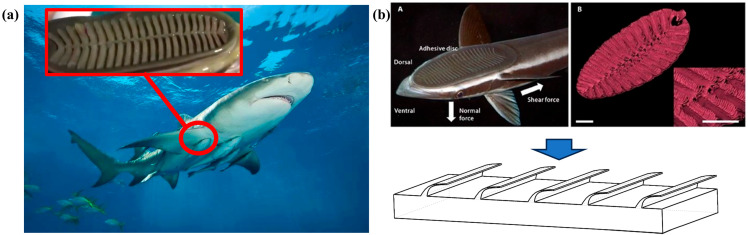
Characterization of remora suckerfish sucker structure and adsorption mechanism study. (**a**) Adsorption state of remora suckerfish and shark (The red part in the middle shows a close-up of the gills of a remora suckerfish.), and (**b**) remora suckerfish sucker structure and three-dimensional schematic diagram.

The sucker is a specialization of the dorsal fin, which has evolved over time to form an elliptical structure, occupying the head to the front of the body, containing 16–28 thin layers with a central longitudinal pleat which can form a strong vacuum adsorption force, as shown in Figure 1a. The edge of the suction cup consists of a ring of soft lip-like tissue, similar to the soft transition zone where a rubber band is combined with a disk, which is both thin and elastic to better fit the surface of the adsorbent object and play a sealing role in preventing water from entering the gap between the suction cup and the adsorbent object.

In this study, the macroscopic convexity of its fin surface was obtained via a literature review and three-dimensional scanning (e.g., Figure 1b). The structure was scaled up proportionally and applied to the surface of the bionic coated tape. After pressure compression, the tape enables high-uniformity material application and prevents the uneven polymer deposition caused by gravity after winding.

### 2.2. Study of Bionic Coating Mechanism of High-Strength Fiberboard Tape

**Figure 2:**
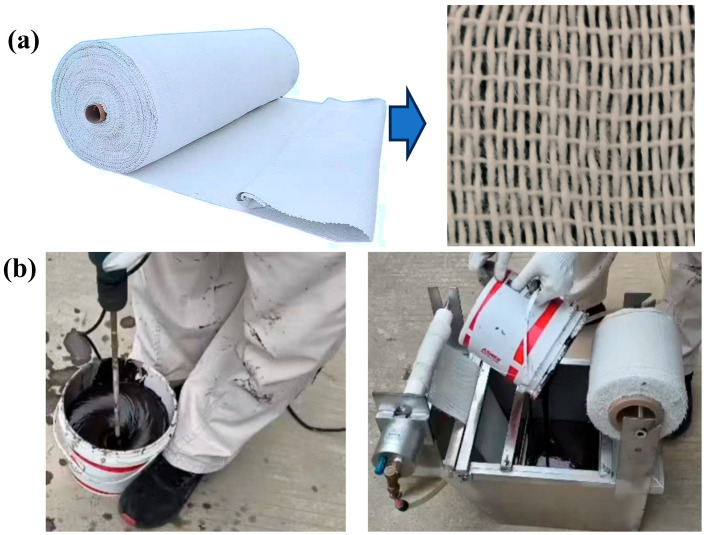
High-strength fiberboard tape. (**a**) Appearance and structure of fiberboard tape, and (**b**) polymer material coating process.

Fiberboard tape was used as the base material of the nano-polymer–epoxy composite system (Figure 2a), and its three-dimensional network structure provided an ideal mechanical anchoring interface for the resin matrix to ensure the formation of a uniform and stable composite structure of the material during the dynamic coating process. During application, the self-developed hybrid polymer—solvent-free epoxy reinforcement material—is employed, comprising modified epoxy resin, filler, and a compounded curing agent. The material is composed of modified epoxy resin, filler, and a compound curing agent. The corresponding proportion of the material is stirred uniformly and poured into the material storage pool, which can be uniformly dipped and coated with polymer material on both sides through the control of dipping and coating of the control parts of the winding equipment. The high-strength fiberboard tape thus obtained has a large amount of polymer material attached to the surface, and when it is wound on the surface of the pipe to be reinforced, it will be surface-dried within one hour and completely dry within 24 h, as in Figure 2b.

However, due to the effect of gravity, the material is unevenly distributed. In addition, the interfacial bonding behavior between the fiberboard tape and the resin matrix is significantly affected by the loads of the complex marine environment, including wave impact, hydrostatic pressure, and hydrodynamic vibration, resulting in a non-uniform distribution of the interfacial bonding strength and a weakening of the bond in local areas.

In this study, we propose a biomimetic interface design strategy to construct multi-stage biomimetic adhesion units on the surface of fiberboard tapes by mimicking the micro-nanostructural characteristics of remora suckerfish suction cups. Combined with the dynamic pressure field control technology, a dynamic balance of the interfacial stress is realized during the curing process of the composite system, which effectively suppresses the uneven coating thickness caused by gravitational segregation and fluid shear and ensures the stable and reliable interfacial performance of the composite structure in service.

### 2.3. Discrete Meta-Analytical Modeling of Nano-Polymer Materials for Epoxy Materials

#### 2.3.1. Discrete Element Modeling Study

**Figure 3:**
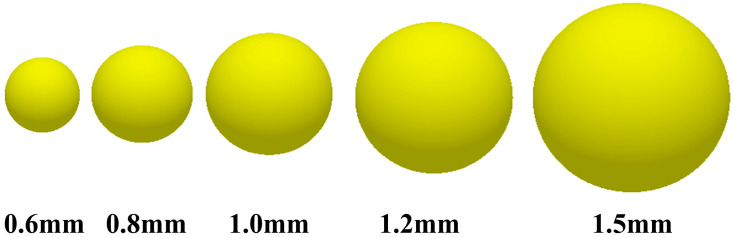
Discrete element model of particles.

In the discrete element modeling process, if the geometric particle size is not properly selected, not only can it not accurately simulate the real behavior of the material but it also may lead to the waste of computational resources or the bias of computational results. Therefore, it is a necessary prerequisite to establish an effective discrete element model by fully considering the highly viscous characteristics of the material and reasonably determining the size and shape of the geometric particles. The single-sphere model with radii of 0.6 mm, 0.8 mm, 1 mm, 1.2 mm, and 1.5 mm is selected as the research object, as shown in Figure 3. The single-sphere model has the advantages of having a simple structure and clear parameters, which are easy to calculate and analyze in discrete element simulation and also can reflect the basic properties of material particles to a certain extent. By constructing this series of single-sphere models with different sizes, the effect of geometric size changes on the simulation results of the high-adhesion behavior of materials can be systematically investigated.

The discrete element simulation and analysis software utilized in this paper is EDEM (2020). EDEM is a general-purpose CAE software used to simulate and analyze particle handling and production operations, which simulates the processes of the collision, flow, and accumulation of particles.

The JKR model is a theoretical model describing the mechanics of adhesion contact, which was proposed by Johnson, Kendall, and Roberts in 1971 [27], for adhesion phenomena arising from the mutual contact of soft materials. In contrast to the traditional Hertz contact theory, the JKR theory takes into account the effect of surface energy on the contact area and considers that attractive forces are generated at the surface of the material, leading to an increase in the contact area. In the JKR model implemented in EDEM, the normal contact force (or adhesion force) in the JKR model is defined as follows:

(1) $$F_{n}=\frac{4E^{*}a^{3}}{3R^{*}}-{\left(8\pi \Gamma E^{*}a^{3}\right)}^{\frac{1}{2}}$$

where *E*×, *R*×, *Γ* and a are the relative elasticity, relative radius, interfacial surface energy (also known as work of adhesion), and contact radius (as described in Thornton [28]), respectively. This is not the same as the EDEM contact radius. In this implementation of the model, the adhesive force depends on the interfacial surface energy and the relative approach (negative) at which the contact breaks. These are defined by the following generalized equations:

(2) $$\Gamma =\gamma_{1}+\gamma_{2}-\gamma_{1,2}$$

(3) $$\alpha_{f}={\left(\frac{3F_{nc}^{2}}{16R^{*}E^{*2}}\right)}^{\frac{1}{3}}$$

where *γ*_1_ and *γ*_2_ are the surface energies of the two spheres, *γ*_1,2_ is the interfacial surface energy, and αf is the relative approach where the contact breaks. For the special case where two spheres of the same material come into contact, the interfacial surface energy is zero, *γ*_1,2_ = 0, and the interfacial surface energy becomes Γ = 2*γ*.

The relative approaching distance, α, (also known as contact overlap) and the pull-up force are defined by the following equations:

(4) $$\alpha =\frac{a^{2}}{R^{*}}-{\left(\frac{2\pi \Gamma a}{E^{*}}\right)}^{\frac{1}{2}}$$

(5) $$F_{nc}=\frac{3}{2}\pi R^{*}\Gamma$$

where a is the normal overlap between particles. For contacts between spheres of the same material, Γ = 2*γ*, and so Equations (1) and (4) can be rewritten as follows:

(6) $$F_{n}=\frac{4E^{*}a^{3}}{3R^{*}}-4{\left(\pi \gamma E^{*}a^{3}\right)}^{\frac{1}{2}\;}$$

(7) $$\alpha =\frac{a^{2}}{R^{*}}-{\left(\frac{2\pi \Gamma a}{E^{*}}\right)}^{\frac{1}{2}}$$

#### 2.3.2. Simulation Model Parameter Testing and Calibration

**Figure 4:**
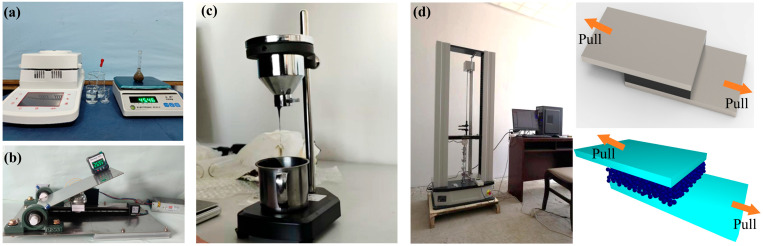
Simulation of basic parameter testing: (**a**) halogen analyzer; (**b**) coefficient of static friction tester; (**c**) coefficient of rolling friction testing process; and (**d**) JKR parameter testing and calibration process.

The intrinsic parameter tests are carried out for polymer materials. The results of the previous tests show that the intrinsic parameters of polymer materials are not correlated with the variable parameters of the material model, while the contact parameters are highly correlated with the variable parameters of the material model. The moisture content of the polymer material was determined with the help of a halogen moisture detector. The Poisson’s ratio of polymer material was determined with reference to the ASAE standard [29,30]. The density of the polymer material was measured with the help of the gravimeter method. The modulus of elasticity of the polymer material was measured by compression test using an electronic universal testing machine [31]. The coefficient of static friction between the polymer material, stainless steel, and the bionic fiberboard tape was measured using the slope method [32,33,34]. The coefficient of recovery between the polymer material, stainless steel, and the bionic fiberboard tape was measured by a drop test and a pendulum test, respectively, using a high-speed video camera [35,36], and the coefficient of rolling friction between the polymer material, stainless steel, and the bionic fiberboard tape was calibrated using a funnel test. The steel plates or fabrics bonded together through polymer materials were stretched by a tensile testing machine and the JKR parameters were obtained by means of simulation calibration, as shown in Figure 4. The corresponding parameters obtained are shown in Table 1.

### 2.4. Test and Simulation Verification

#### 2.4.1. Simulation Model Validation Test

Based on the discrete element model, the volumetric density test is performed along with the droop test. The volumetric density test is a test used to determine the mass per unit volume of a material in a certain volumetric state. It can be used to assist discrete element modeling software to determine the simulation accuracy of the simulated particles. The drop test is used to determine the adhesion of highly adhesive materials to other materials, and to determine the accuracy of the contact parameters and the adhesion model.

**Figure 5:**
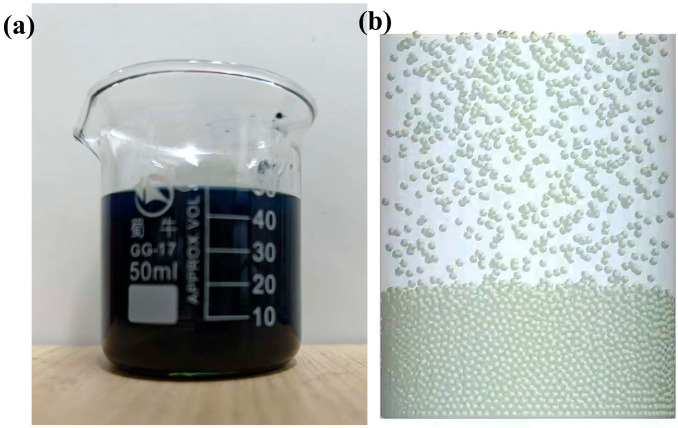
Volumetric density test. (**a**) Schematic of the actual test; (**b**) volumetric density simulation test.

The volumetric density test was conducted using a 50 mL beaker, in which the polymer material was added to the beaker to determine the volume, and additionally the mass value of the polymer material was mapped to determine the final volumetric density parameters. The simulation test was performed by establishing a beaker model of the same size through the particle factory to produce the corresponding mass of particles, changing the diameter of the particles to determine the volume produced by different diameter particles, and selecting the appropriate particle diameter. Each test and simulation test were conducted three times, respectively. The specific process is shown in Figure 5.

**Figure 6:**
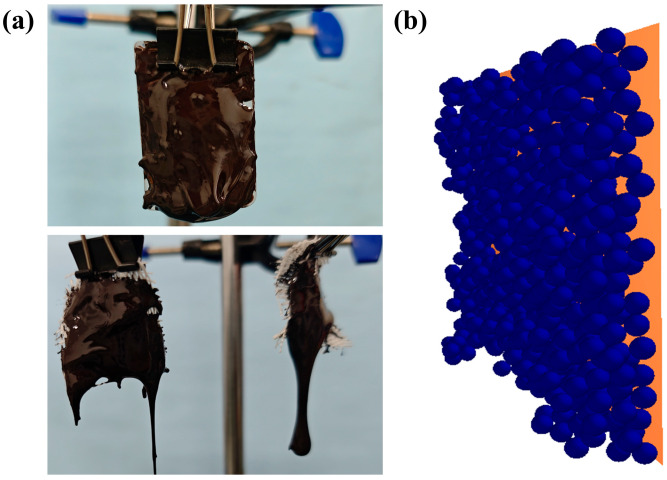
Drip droop test. (**a**) Actual equipment for the drop test (galvanized steel/fiberboard tape + polymer material, suspended to record drops over a 5 min period); (**b**) simulated test set up (90° rotating plane, observation of particle residual mass).

The drip droop test uses a galvanized steel plate with fiberboard tape of 30 mm × 30 mm area, which is placed on a flat surface, with 2 g of polymer material then applied to its surface. It is hung up and the dropping within 5 min is recorded. Different contact materials were replaced to determine the final residual mass, as well as the shape of the drops. In the simulation test, a plane of corresponding size was created with a particle project to generate particles of corresponding mass. The rotating vice was set up to form a 90-degree rotation, and the residual mass was recorded to observe the drooping of the particles. Each test and simulation test were conducted three times, respectively. The specific process is shown in Figure 6.

#### 2.4.2. Optimization Test of Bionic Structure Combination

**Figure 7:**
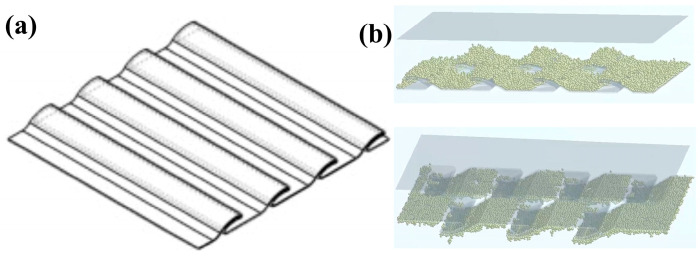
Bionic structure combination optimization test. (**a**) Schematic diagram of the bionic structure, and (**b**) simulation test process.

After determining the accuracy of the bionic model, the corresponding bionic structure combination optimization test was conducted. In order to confirm the optimal results, a one-factor test was conducted in the preliminary stage to determine the main variable parameters in the bionic parameters, which are transverse frequency, strip depth, and longitudinal frequency, as shown in Figure 7. A multi-factor simulation analysis test was carried out for the above problems, and the test factors and specific level values are shown in Table 2. During the test, a simulation fiberboard tape with a length of 200 mm and a width of 200 mm was established, and the corresponding undulating stripe situation was established on its surface. From this, 50 g of polymer material was generated, rotated 180 degrees, and the particle falling situation of the particles within 3 min was determined.

#### 2.4.3. Curing Test

**Figure 8:**
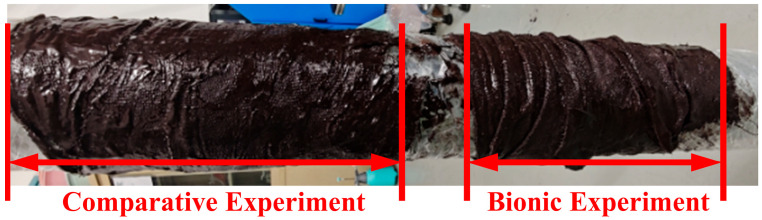
Curing test.

Through the bionic structure combination optimization test to determine the optimal bionic structure, the corresponding existing structure and the bionic structure of the control test were found, from which the final operating effect was determined. In the test process a cast iron pipe with a diameter of 150 mm is used, and the original fabric and the bionic fabric are selected to be wound with polymer material, which is static for 24 h after winding, and then a compression test is carried out by using a tensile testing machine to determine the final operating effect. The test process and the machine are shown in Figure 8.

## 3. Results and Discussion

### 3.1. Simulation Model Validation Test Results and Discussion

**Figure 9:**
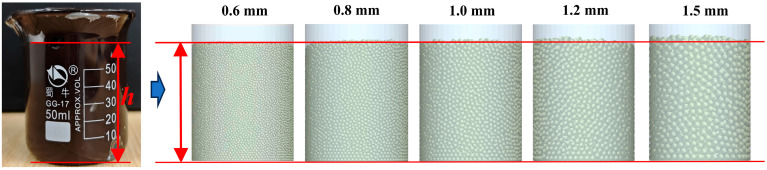
Volumetric density test results.

The volumetric density simulation results shown in Figure 9 reveal the significant effect of particle geometry on the accuracy of the discrete element model. In the particle models with radii of 0.6 mm, 0.8 mm, and 1 mm, the simulation results show a high degree of consistency and good agreement with the actual test data, and the error range is controlled in the acceptable engineering interval (≤5%). In contrast, the simulation results for the 1.2 mm and 1.5 mm radii were internally consistent but deviated significantly from the measured values (≥12% error). This discrepancy stems from the “scale effect” induced by particle size increase as when the particle size exceeds 1 mm, gravity-driven settling becomes the primary factor governing the stacking process, overriding surface forces. Meanwhile, limitations in the contact force algorithms of existing discrete element models—specifically in geometrically describing void distributions between large particles—amplify porosity calculation errors. In addition, the stacking structure of the large-particle system is more susceptible to boundary conditions, and the periodic boundary conditions used in the simulation differ equivalently from the container constraints of the actual test, further exacerbating the density prediction error. Therefore, in order to improve the simulation accuracy and reduce the computational difficulty of the test process, a particle radius of 0.8 mm was selected.

**Figure 10:**
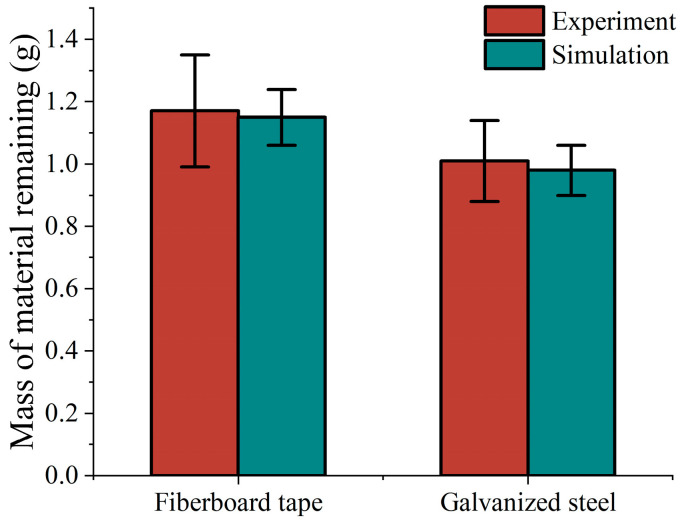
Comparison of residual mass of polymer material in drop pendant test.

As shown in Figure 10, the figure visualizes the comparison between the experimentally measured and simulated residual mass of the polymer material under the conditions of two different materials, namely, fiberboard tape and a galvanized steel sheet. As clearly shown in the figure, the test results for the fiberboard tape and galvanized steel align closely with the experimentally measured residual mass of the polymer material, which is slightly higher than the simulated value. At the same time, the experiments present a larger error range than the simulation results. This result shows that the parameters of rolling friction, sliding friction, and JKR used in the study and simulation have high accuracy. These parameters can accurately reflect the actual situation, which provides a reliable support for further research on the properties of polymer materials on different material surfaces.

Analyzing from the theoretical level, the precise setting of intrinsic parameters is the foundation of reliable simulation results. These parameters accurately portray the material’s own intrinsic properties, including density, elastic modulus, etc., which lay a solid material foundation for the simulation. The accurate selection of contact parameters is the key to the success of the model, which describes the interaction mechanism between particles in detail and enables the simulation to truly simulate the complex mechanical behavior between particles. The accurate expression of the JKR adhesion behavior effectively captures the adhesion phenomenon triggered by the surface tension and van der Waals force of the particles at a small scale, which is crucial for the simulation of highly viscous materials.

### 3.2. Bionic Structure Combination Optimization Test Results and Discussion

The results of the bionic structure combination optimization test are shown in Table 3, and the F-value of the model is 3.87 which indicates that the model is significant. There is only a 4.41% probability that such a large F-value will be generated due to noise. When the *p*-value is less than 0.0500, it indicates that the model term is significant. In this case, A, B, C, and B^2^ are significant model terms. When the *p*-value is greater than 0.1000, it means that the model term is not significant. If there are a large number of non-significant model terms (that do not contain the terms needed to support the hierarchy), model reduction may be able to optimize the model. The F-value for the misfit test is 1.38, which means that misfit is not significant relative to pure error. There is a 36.94% probability of having such a large F-value for misfit due to noise. The fact that the misfit is not significant proves the validity of the model. Therefore, based on the above issues, the basic equation and the specific impact results were determined.

By analyzing the data, the corresponding Equation (8) can be determined:

(8) $$M=1.42+0.2810A+0.2966B+0.2562C+0.0218B^{2}$$

Longitudinal frequency, bar depth, and transverse frequency all positively affect drop quality. Specifically, each unit increase in longitudinal frequency positively affects drop quality to some extent in a linear manner; transverse frequency also affects drop quality in a positive linear relationship, but the linear effect of longitudinal frequency is stronger than that of transverse frequency. In particular, the mechanism of bar depth is more complex as it exists as a quadratic term in the model. This means that as the bar depth increases, its effect on the drop quality is not simply linearly increasing but will be accelerated and strengthened with an increase in the bar depth value; highlighting that the effect of the bar depth on the drop quality has the characteristic of nonlinear enhancement.

Therefore, in order to determine the basic parameters for the polymer drop, a longitudinal frequency of 2, a bar depth of 11.822 mm, and a transverse frequency of 2 are selected as the optimal combination, and the effectiveness of the optimal model is determined through subsequent tests.

### 3.3. Curing Test Results and Discussion

**Figure 11:**
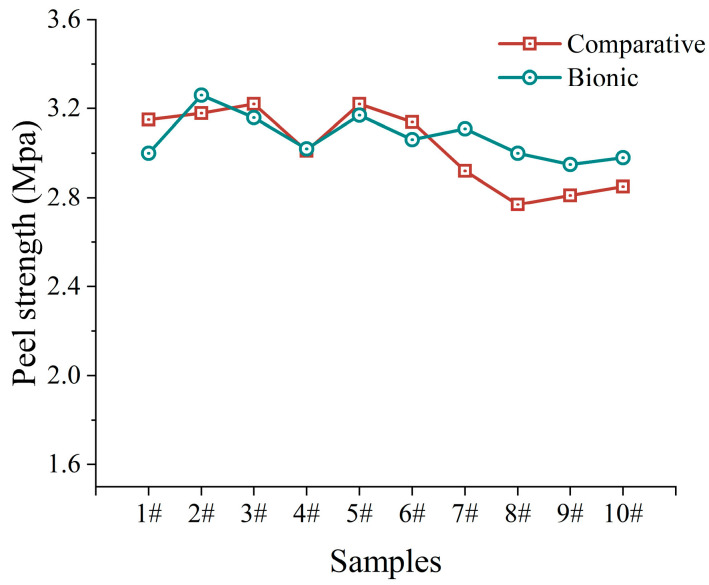
Peel strength results for different materials in the curing test.

As shown in Figure 11, this is the presentation of the results of the curing test. In this test, samples were collected in the opposite direction of gravity for both the control group and the bionic mechanism group. The purpose of this sampling method is to accurately investigate the effect of gravity on the peel strength of the cured samples. From the graphical data, the performance of the control samples shows a distinctive feature. The peel strength of the top sample of the control group was relatively low due to the effect of gravity. Not only that, but the peel strength also fluctuates greatly from samples 1# to 10# as the value fluctuates significantly, reflecting the poor consistency of performance between samples. On the other hand, the performance of the bionic structure group is impressive. The peel strength of the bionic structure is relatively stable and balanced, with small differences in the values of the samples, and the stability of the bionic structure is improved by about 43.29% compared with the comparison test. This stable characteristic makes the overall strength highly suitable for the actual production requirements, and in the actual production it can effectively avoid the unstable product quality caused by the strength fluctuation and other problems, which shows the significant advantages and potential of the bionic structure in the application of material curing.

## 4. Conclusions

In order to meet the special needs of the preventive rehabilitation of oil and gas well pipelines, this study is based on the principle of bionics to dissect the geometric structure of remora suckerfish suckers and combine them with fiberboard tape to determine the optimal combination results by discrete element model. The results are summarized as follows:

1. Geometrical analysis of remora suckerfish based on bionic principle and combination of remora suckerfish with fiberboard tape to provide the bionic basis for novel structural design.
2. Construction of the particle model of a polymer material without solvent epoxy reinforcement by discrete element model to determine the relevant parameters. The accuracy of the model is verified by solvent density test and drop test, and then the optimal combination of parameters of the bionic structure is obtained with the help of multi-factor test simulation analysis.
3. The bionic coating structure, inspired by remora sucker geometry, was developed through three-dimensional scanning (0.02 mm accuracy) and proportional scaling. Optimization of longitudinal pleat parameters (height: 1.2 mm and spacing: 3.5 mm) achieved a 43.29% improvement in coating thickness uniformity. The gravitational segregation-induced density gradient was reduced from 0.12 g/cm^3^ to below 0.04 g/cm^3^, effectively addressing thickness inconsistency issues in traditional materials which are caused by gravitational effects.
4. Multi-factor response surface analysis shows that the bionic structure decreased the standard deviation of interfacial bond strength from 0.21 MPa to 0.12 MPa, with the average strength increasing by 0.2 MPa (from 2.8 MPa to 3.0 MPa)—meeting the safety threshold of ≥2.5 MPa for pipeline repair. Drop tests confirmed that coating thickness unevenness was reduced from 7–10% to within 3%, validating the structure’s inhibition of fluid shear effects.
5. Determined the bionic structure combination optimization results through a multi-factor test, determined that the longitudinal frequency, bar depth, and transverse frequency have a positive effect on the quality of the drop, and thus a longitudinal frequency of 2, a bar depth of 11.822 mm, and a transverse frequency of 2 were selected as the optimal combination of results.
6. The comparison test between the bionic structure and the original structure confirms that the bionic structure has good stability and effectively avoids problems such as a lack of strength, and its stability is improved by about 43.29% compared to the original structure.

The study successfully solved the problem of uneven coating thickness caused by gravitational segregation and fluid shear, guaranteed the stable and reliable interfacial properties of composite structures during service, and provided strong support for the practical application of related materials in the preventive repair of oil and gas well pipelines. In the future, it will promote the upgrading of the maintenance strategy of oil and gas pipelines from “passive response” to “active prevention” and form the core technological support for the resilience of energy infrastructure.

## Figures and Tables

**Table 1:** Parameters of the simulation model.

Parameters	Value	Parameters	Value
Density kg/m^3^		Polymer material–galvanized steel	0.020
Polymer material	1677	Polymer material–fiberboard tape	0.050
Galvanized steel	7865	Coefficient of static friction	
Fiberboard tape	1073	Polymer material–polymer material	0.947
Poisson’s ratio		Polymer material–galvanized steel	0.976
Polymer material	0.31	Polymer material–fiberboard tape	0.903
Galvanized steel	0.30	Coefficient of rolling friction	
Fiberboard tape	0.41	Polymer material–polymer material	0.477
Shear modulus Pa		Polymer material–galvanized steel	0.498
Polymer material	1.773 × 10^6^	Polymer material–fiberboard tape	0.406
Galvanized steel	7.900 × 10^10^	JKR Energy Density J/m^2^	
Fiberboard tape	1.210 × 10^8^	Polymer material–polymer material	4.57
Coefficient of restitution		Polymer material–galvanized steel	3.81
Polymer material–polymer material	0.030	Polymer material–fiberboard tape	2.11

**Table 2:** Multi-factor test combinations and levels for optimization of bionic structure combinations.

Level	−1	0	1
Longitudinal frequency	1	2	3
Strip depth	10	14	18
Transverse frequency	2	4	6

**Table 3:** Optimization test results of bionic structure combination.

Source	Sum of Squares	df	Mean Square	F-Value	*p*-Value	
Model	2.97	9	0.3296	3.87	0.0441	Significant
A—Longitudinal frequency	0.6317	1	0.6317	7.41	0.0297	Effective
B—Strip depth	0.7037	1	0.7037	8.26	0.0239	Effective
C—Transverse frequency	0.5250	1	0.5250	6.16	0.0421	Effective
AB	0.0410	1	0.0410	0.4814	0.5102	-
AC	0.0076	1	0.0076	0.0895	0.7735	-
BC	0.0028	1	0.0028	0.0327	0.8616	-
A^2^	0.1622	1	0.1622	1.90	0.2102	-
B^2^	0.7359	1	0.7359	8.63	0.0218	Effective
C^2^	0.0736	1	0.0736	0.8636	0.3837	-
Residual	0.5966	7	0.0852			
Lack of fit	0.3036	3	0.1012	1.38	0.3694	Not significant
Pure error	0.2929	4	0.0732			
Cor total	3.56	16				

## Data Availability

The raw data supporting the conclusions of this article will be made available by the authors on request.
